# Shengdihuang Xiyangshen Zhimu Cangzhu Formula Improves Hepatic Glycogen Synthesis via the JNK/c‐Jun/IRS1/GSK3*β* Signaling Pathway

**DOI:** 10.1155/jdr/6019622

**Published:** 2026-04-15

**Authors:** Kewen Qu, Mei Li, Jinxu Sun, Shangping Wu, Xinyu Liu, Kanglin Liu, Shuxin Wu, Xiangyu Guo

**Affiliations:** ^1^ Department of Endocrinology, Dongfang Hospital, Beijing University of Chinese Medicine, Beijing, China, bucm.edu.cn

**Keywords:** c-Jun N-terminal kinase (JNK), diabetes mellitus (DM), hepatic glycogen synthesis, inflammatory factors, network pharmacology, Shengdihuang Xiyangshen Zhimu Cangzhu (SXZC) formula

## Abstract

Diabetes mellitus (DM) is one of the major chronic diseases endangering human health worldwide, and disorders of hepatic glucose metabolism are its main feature. Glucose homeostasis is maintained by hepatic glycogen synthesis and catabolism, glycolysis, and gluconeogenesis. Inflammation can inhibit glycogen synthesis and cause persistent hyperglycemia via the c‐Jun N‐terminal kinase (JNK) signaling pathway. Shengdihuang Xiyangshen Zhimu Cangzhu (SXZC) formula is a botanical medicine complex with hypoglycemic effects, and it is not clear whether the SXZC formula could improve glucose metabolism in the liver. In this study, we are aimed at investigating the effect of the SXZC formula on hepatic glucose metabolism. The SXZC formula was administered to db/db mice for 4 weeks. Three concentration gradients of the SXZC formula were administered: a high‐dose group (5.96 g·kg^−1^·d^−1^), a medium‐dose group (2.98 g·kg^−1^·d^−1^), and a low‐dose group (1.49 g·kg^−1^·d^−1^). The potential signaling pathways were identified by network pharmacology, subsequently validated via molecular docking and kinetic simulations, as well as molecular experiments. It was found that the SXZC formula reduced blood glucose levels, inflammatory and phosphorylation levels of JNK, c‐Jun, and insulin receptor substrate 1 (IRS1), and promoted glycogen synthase kinase‐3 β (GSK3*β*) phosphorylation and hepatic glycogen synthesis. We indicated that the SXZC formula alleviates hepatic inflammation and promotes hepatic glycogen synthesis by the JNK/c‐Jun/IRS1/GSK3*β* signaling pathway.

## 1. Introduction

Diabetes mellitus (DM) is a chronic endocrine disease characterized by disorders of glucose and lipid metabolism [1]. The liver is the primary organ responsible for blood glucose homeostasis [2], which accounts for about 90% of endogenous glucose production in the body [3]. Impaired hepatic glucose metabolism plays an important role in dysglycemia.

Glycogen synthesis is regulated by the insulin (INS) signaling pathway, together with glycogenolysis, gluconeogenesis, and glycolysis, participating in the precise regulation of glucose homeostasis [4, 5]. When excessive ectopic accumulation of fat occurs in the liver, it would cause an inflammatory response and insulin resistance (IR) [6]. Inflammatory factors (IFs) include tumor necrosis factor‐*α* (TNF‐*α*), interleukin 1 *β* (IL‐1*β*), and interleukin 6 (IL‐6) generated by endoplasmic reticulum stress and oxidative stress in “ballooned” hepatocytes, activating the c‐Jun N‐terminal kinase (JNK)/c‐Jun pro‐inflammatory signaling pathway. This affects insulin receptor substrate 1 (IRS1)–mediated phosphatidylinositol 3‐kinase (PI3K)/protein kinase B (Akt) signaling, which directly leads to downregulation of glycogen synthase kinase‐3 (GSK‐3) and inhibition of glycogen synthesis [7]. Simultaneously, the abnormal accumulation of IFs exacerbates IR and forms a vicious circle between disordered glycolipid metabolism and the inflammatory response [8, 9].

Botanical medicine has shown potential in improving glycolipid metabolism [10]. Traditional Chinese medicine (TCM) formulations possess unique advantages in treating metabolic disorders with multiple causative factors through synergistic interactions between multiple herbs [11]. Shengdihuang Xiyangshen Zhimu Cangzhu (SXZC) formula is an herbal medicine originating from the Qingdanzhixiao formula. SXZC formula is made up of these four herbs in specific proportions according to the principles of TCM theories. Studies have shown the therapeutic effects of these herbs on hypoglycemia. Shengdihuang (*Rehmanniae Radix*) extract ameliorated hyperglycemia and IR by upregulating transient receptor potential cation channel subfamily V member 1 (TRPV1) and downregulating stearoyl‐CoA desaturase 1 (SCD1) expression levels in streptozotocin (STZ)–induced SD rats and HepG2 [12]. In HepG2 cells, ginsenoside Rg1 of xiyangshen (*American ginseng*) potentiated the phosphorylation of AMP‐activated protein kinase (AMPK)/Forkhead box protein O1 (FoxO1) in a time‐ and concentration‐dependent manner, concurrently repressing gluconeogenic enzyme expression and enhancing glucose uptake [13]. Following treatment with Zhimu (*Anemarrhenae Rhizoma*) polysaccharides, STZ‐induced diabetic mice (C57BL/6J) exhibited a dose‐dependent decrease in *α*‐glucosidase activity, which contributed to improved glycemic control [14]. In vitro, atractylenolide II from Cangzhu (*Rhizoma Atractylodis*) showed an inhibitory effect on the diacylglycerol kinase *θ* (DGK*θ*)/AMPK/peroxisome proliferator–activated receptor‐*γ* coactivator 1*α* (PGC1*α*)/uncoupling protein 1 (UCP‐1) signaling pathway, which ameliorated obesity‐induced disorders and reduced body weight [15]. Although the effects of individual herbs are better defined, it is not clear whether the SXZC formula could exert its hypoglycemic effect by regulating hepatic glucose metabolism.

In this study, we conducted an in vivo experiment to verify the therapeutic effect of SXZC formula. Network pharmacology, molecular docking, and kinetic simulation methods were applied to reveal its potential mechanism. We found that the SXZC formula could inhibit inflammation and promote glycogen synthesis through the JNK/c‐Jun/IRS1/glycogen synthase kinase‐3*β* (GSK3*β*) pathway, thereby exerting a hypoglycemic effect.

## 2. Materials and Methods

### 2.1. Chemicals and Reagents

The primary antibodies, including *β*‐actin (4970S, 1: 1000), SAPK/JNK (67096S, 1: 1000), phospho‐SAPK/JNK (4668S, 1: 1000), phospho‐IRS‐1 (2381S, 1: 1000), c‐Jun (9165S, 1: 1000), phospho‐c‐Jun (3270 T, 1: 1000), and phospho‐GSK3*β* (5558 T, 1: 1000), were purchased from Cell Signaling Technology, United States. IRS1 rabbit mAb (AFRM9574, 1: 2000) was purchased from Afantibody, China. GSK3*β* recombinant monoclonal antibody (82061‐1‐RR, 1: 5000) was purchased from Proteintech Group, United States.

*Rehmanniae Radix*, *American ginseng*, and *Anemarrhenae Rhizoma* were procured from Beijing Renwei Chinese Herbal Medicine Slices, China. *Rhizoma Atractylodis* was sourced from Sifang Pharmaceutical, China. The herbal mixture consisted of *Rehmanniae Radix*, *American ginseng*, *Anemarrhenae Rhizoma*, and *Rhizoma Atractylodis* in a ratio of 3:1:2:1. We added eight times the volume of water, soaking for 1 h, decocting for 1.5 h. Then, the mixture was decocted again with six times the volume of water for a further 1.5 h. The decoction was vacuum‐dried to obtain a water extract, which was further pulverized and sieved into dry powder. Finally, the dry extract powder was mixed with dextrin to prepare the final product. The yield of the dry extract was approximately 32.72%.

All other reagents were of commercial grade and obtained from various reputable suppliers.

### 2.2. Animals and Treatment

Seven‐week‐old male db/m and db/db mice were purchased from Cavens Laboratory Animal, China. All mice were reared in the animal center of Beijing University of TCM with access to normal chow and water, ad libitum. The housing environment was maintained at 22°C–24°C, 50%–60% relative humidity, and a 12:12 h light: dark cycle.

After 1 week of acclimatization, 35 db/db mice were evenly allocated to five groups via block randomization according to their body weight and fasting blood glucose (FBG) levels: model group, Met (metformin) (0.2 g·kg^−1^·d^−1^) group, SXZC‐H (high dose, 5.96 g·kg^−1^·d^−1^) group, SXZC‐M (medium dose, 2.98g·kg^−1^·d^−1^) group, and SXZC‐L (low dose, 1.49 g·kg^−1^·d^−1^) group (*n* = 7 per group). Additionally, seven db/m mice were set as normal group. Administration was as follows: Normal and model group received deionized water (1 mL·100 g^−1^·d^−1^) by gavage; other groups received corresponding drugs at the same volume and frequency for 4 weeks.

During the experiment, body weight, FBG (U‐Amgen blood glucose meter and test strips, Bayer, Germany, 59400862136) levels were measured after 8 h of fasting at Weeks 0, 1, 2, 3, and 4. Protocol of oral glucose tolerance test (OGTT) were as follows: At Week 4, mice were fasted for 12 h, and gavaged with glucose (2 g/kg). Blood glucose was measured at 0, 30, 60, 90, and 120 min, and the area under the curve (AUC) was calculated.

After 4 weeks of continuous gavage, all mice were fasted for 12 h. Under deep anesthesia, blood was collected from the retro‐orbital venous plexus, followed by euthanasia via cervical dislocation. The blood was centrifuged to collect serum, which was stored at −20°C for biochemical analysis. A portion of liver tissue was fixed in 4% paraformaldehyde (Solarbio, China, P1110) for histopathological examination. The remaining portion was flash‐frozen in liquid nitrogen and stored at −80°C for subsequent molecular experiments.

Serum levels of INS and glycated serum protein (GSP) were detected using a fully automated biochemistry analyzer (Mindray, China, BS‐420).

### 2.3. Enzyme‐Linked Immunosorbent Assay (ELISA)

Liver tissue samples were used to detect IL‐6, tumor necrosis factor‐α (TNF‐*α*), and interleukin 1 β (IL‐1*β*) levels via ELISA kits (Elabscience, China, E‐EL‐M0044, E‐EL‐M3063, E‐EL‐M0037). A total of 100 *μ*l of sample or standard was added to each well of the microplate and incubated at 37°C. Subsequently, the following reagents were sequentially added: biotinylated detection Ab, HRP conjugate, substrate reagent, and stop solution. Finally, the optical density (OD) at 450 nm for each well was measured using an enzyme label analyzer (Promega GloMax Discover System, USA, GM3000).

### 2.4. Liver Histopathology

#### 2.4.1. Hematoxylin–Eosin (HE) Staining

Mice liver tissues were fixed in 4% paraformaldehyde solution for 48 h, then dehydrated through gradient alcohol and xylene, embedded in paraffin, and sectioned at 3‐*μ*m thickness (Neutral Mounting Medium, Sinopharm, China, 10004160). Sections were stained with hematoxylin–eosin (HE) (hematoxylin and eosin, Raisedragon, China, LMDS‐C‐S‐2025, LMDS‐C‐Y2025) to examine tissue histomorphology through an optical microscope.

#### 2.4.2. Periodic Acid‐Schiff (PAS) Staining

Liver paraffin sections were dewaxed in water, rinsed with distilled water, and soaked in 70% alcohol. The sections were immersed in periodate alcohol solution for 10 min (17°C–20°C), rinsed in 70% alcohol. We put them into a reducing solution for 1 min (17°C–20°C), and rinse again with 70% alcohol. After incubation in colorless saline‐based magenta solution for 60–90 min and rinsing with running water for 10 min, the nuclei were stained with hematoxylin for 3–5 min, differentiated in 1% hydrochloric acid alcohol, dehydrated, and sealed (PAS Staining Kit, Raisedragon, China, LMDS‐C‐Y2507). The distribution of liver glycogen was observed under an optical microscope.

### 2.5. Reverse Transcription–Quantitative Polymerase Chain Reaction (RT‐qPCR)

Total ribonucleic acid (RNA) was extracted from liver tissue (SteadyPure Universal RNA Extraction Kit, Agbio, China, AG21017). Complementary deoxyribonucleic Acid (cDNA) was synthesized from total RNA, and the target gene was subsequently amplified using a reverse transcription kit (Evo M‐MLV One‐Step RT‐qPCR Kit, SYBR method, Agbio, China, AG11732). The PCR reaction conditions were as follows: Reverse transcription at 42°C for 5 min, initial predenaturation at 95°C for 10 s, followed by 40 cycles of amplification (95°C for 5 s and 60°C for 30 s), and final melting curve analysis was executed. Relative mRNA expression levels of G6pc1 and Pck1 were calculated utilizing the 2^−*ΔΔ*Ct^ method, with *β*‐actin as the internal reference. Primer sequences are shown in Table 1.

**Table 1:** Primers used for quantitative RT‐qPCR.

Name	Sequence (5 ^′^ →3 ^′^)
*β*‐actin F	CTCCTGAGCGCAAGTACTCT
*β*‐actin R	TACTCCTGCTTGCTGATCCAC
G6pc1 F	GTCGTGGCTGGAGTCTTGTCA
G6pc1 R	CGGAGGCTGGCATTGTAGATG
Pck1 F	AGTGCCCCATTATTGACCCTG
Pck1 R	ATGATGATCTTGCCCTTGTGT

### 2.6. Western Blot (WB)

Liver tissues were applied for the extraction of total protein (column animal tissue/total cell protein extraction kit, Epizyme, China, PC201). After the protein concentration was evaluated using the BCA protein concentration determination kit (Solarbio, China, PC0020). The lysate containing a protease and phosphatase inhibitor mix (100X, Epizyme, China, GRF103) and SDS‐PAGE protein loading buffer (Solarbio, China, BL502B) were added to adjust all samples to the same density. Subsequently, the proteins were separated by electrophoresis (Servicebio, China, G2149‐1L) in an SDS‐PAGE gel (6%, 7.5%, 8%, 10%, Epizyme, Servicebio, PG210, G2176‐50T, G2177‐50T). Following gel transfer (Servicebio, China, G2148‐1L) onto a PVDF membrane using a Bio‐Rad Protein Blotting System (Bio‐Rad Laboratories, United States, Cat# 1658034, 1658051). The membrane was subjected to blocking with protein‐free rapid blocking buffer (Epizyme, China, PS108P) for 20 min at room temperature, followed by incubation with primary antibodies overnight. And finally, incubate the membrane with goat anti‐rabbit IgG antibody (Cell Signal Tech, United States, 7074S, 1: 3000) for 1 h at room temperature. Protein bands were detected with ECL chemiluminescent substrate (Abclonal, China, RM02867), and protein expression was analyzed and quantified using ImageJ software. The membrane was washed with TBST buffer (Servicebio, China, G0004‐1 L) and stripped with stripping buffer (Epizyme, China, Cat. No. PS107).

### 2.7. Network Pharmacology Analysis

Active components of SXZC formula were screened from TCMSP (https://www.tcmsp-e.com/id = 43) (OB ≥ 30*%*, DL ≥ 0.18) and herb databases (Lipinski’s rule of five) (http://herb.ac.cn/) [16–18]. The active ingredients screened by the two websites were deduplicated and integrated. The simplified molecular input line entry system (SMILES) name or structure was obtained from PubChem database [19] (https://pubchem. Ncbi.nlm. https://pubchem.ncbi.nlm.nih.gov/) or TCMSP database. Then imported into SwissADME website (http://www.swissadme.ch/) for secondary screening [20, 21]. Using SwissTargetPrediction [22] (http://swisstargetprediction.ch/) for target prediction (Possibility ≥ 0.1). OMIM database [23] (https://www.omim.org/), GeneCards database [24] (https://www.genecards.org/), and DisGeNet [25] (https://disgenet.com/) were searched with the keyword “Type 2 diabetes mellitus (T2DM)”. Screening principle of OMIM Database: Only select targets corresponding to disease phenotypes. Screening principle of GeneCards: relevance score > 42.2 (screened based on quartiles). Finally, the targets obtained from the three databases are de‐duplicated and integrated.

Venny 2.1.0 [26] was used to identify intersecting targets between targets relevant to SXZC formula and genes associated with T2DM. The intersecting targets were imported into String database to draw a protein–protein interaction (PPI) network (minimum required interaction score = 0.4). The *TSV*‐format file was downloaded and imported into Cytoscape 3.10.0 for network refinement.

### 2.8. Gene Ontology (GO) and Kyoto Encyclopedia of Genes and Genomes (KEGG) Pathway Enrichment Analysis

The obtained component‐disease intersection targets were imported into the Metascape database (https://metascape.org/gp/#/main/step1) [27]. The species selected was human, and GO analysis was conducted based on biological process (BP), molecular function (MF), and cellular component (CC). KEGG pathway enrichment assay was additionally implemented. The outputs were used to generate bubble plots, bar, and pathway charts by an bioinformatics platform (https://www.bioinformatics.com.cn/) [28].

### 2.9. Molecular Docking

Molecular docking was performed to conduct the validation of the core active ingredient and target. We downloaded the 2D or 3D structure (in SDF or mol2 format) of the core active ingredient from PubChem database or TCMSP database. We converted the SDF or mol2 format to PDB format through Open Babel GUI 3.1.1. 3D structure of the protein in PDB format was acquired from RCSB database [29] (http://www.rcsb.org/). Applying Pymol 3.1.4 to remove ligands and water molecules in the target molecules, and the molecules were hydrogenated by AutoDock 1.5.7. After setting the active pocket parameters, molecular docking was performed in AutoDock Vina 1.1.2. The binding activity between the receptor and ligand was evaluated based on the magnitude of the binding energy. Finally, PyMol 3.1.4 was used to visualize the docking.

### 2.10. Molecular Dynamics Simulation

The ligand–receptor complex with the highest binding affinity in molecular docking was selected for molecular dynamics simulations. CHARMM‐GUI [30] (https://charmm-gui.org/) was used to generate the mdp files required for the simulation: CHARMM General Force (CHARMM36m) was used for the protein and small molecule ligand systems. Fit rectangular waterbox to protein size. The total charge of the system was made neutral by adding the corresponding number of sodium, potassium, and chloride ions, where the ratio of potassium ions to sodium ions was 10:1. Molecular dynamics simulations were performed by GROMACS software (2020.6). The system temperature was steadily increased from 0 K to 310.15 K at a fixed volume and constant heating rate. A 125 ps NVT (isothermal and isovolumetric) system simulation was carried out at 310.15 K, which is aimed at ensuring solvent molecules were uniformly distributed in the solvent box. Finally, a 100 ns NPT (isothermal isobaric) system simulation was performed on the composite system. The root mean square deviation (RMSD) and root mean square fluctuation (RMSF) were calculated to assess the stability of protein and ligand binding.

### 2.11. Statistical Analysis

Data were analyzed by SPSS 20.0, with one‐way analysis of variance (ANOVA) for normally distributed data, and Fisher′s least significant difference (LSD) was indeed applied following a nonsignificant ANOVA result. Tamhane′s T2 test was used when the assumption of homogeneity of variances was violated (as indicated by Levene′s test, *p* < 0.05). Nonparametric tests were used for non‐normally distributed data. Data were presented as mean ± standard deviation (mean ± SD). GraphPad Prism 8.0.2 was used to create graphs. Differences were considered statistically significant at *p* < 0.05.

## 3. Results

### 3.1. SXZC Formula Reduces Hyperglycemia in db/db Mice

To test the hypothesis that SXZC formula improves glucose metabolism in a diabetic model, we treated db/db mice with SXZC formula and assessed key metabolic parameters. As shown in Figures 1a, 1d, and 1e, 7‐week‐old db/db mice exhibited high blood glucose levels and continued to have elevated blood glucose during 4 weeks of feeding (Figure 1a). High GSP and INS levels reflect impaired INS‐mediated glucose metabolism in animals. Treatment with Met significantly lowered the elevated blood glucose and INS levels brought about by glucose metabolism disorders. After SXZC formula administration, we also observed reduced FBG, GSP, and INS levels. It suggests that SXZC formula is able to maintain glucose homeostasis and improve the body′s INS sensitivity, which is corroborated by the results of OGTT experiments and AUC (Figure 1c,f). During the experiment, we did not observe the difference in body weight of db/db mice under different treatments (Figure 1b). Taken together, these findings indicate that SXZC formula exerts a hypoglycemic effect in db/db mice, which appears to be time‐ and dose‐dependent.

Figure 1SXZC formula reduces hyperglycemia in db/db mice. (a) Changes of FBG in each group of mice during 0–4 weeks (*n* = 7). (b) Changes of body weight in each group of mice during 0–4 weeks (*n* = 7). (c) Blood glucose at different points of the OGTT experiment (*n* = 7). (d) Changes of GSP in each group of mice at Week 4 (*n* = 7). (e) Changes of INS in each group of mice at Week 4 (*n* = 7). (f) The AUC results obtained from the OGTT experiment (*n* = 7). Met: metformin, SXZC‐H: high dose of SXZC formula, SXZC‐M: medium‐dose of SXZC formula, SXZC‐L: low‐dose of SXZC formula. Compared with the normal group, #*p* < 0.05, ##*p* < 0.01, ###*p* < 0.001. Compared with the model group,  ^∗^ *p* < 0.05,  ^∗∗^ *p* < 0.01,  ^∗∗∗^ *p* < 0.001.

**Figure:**
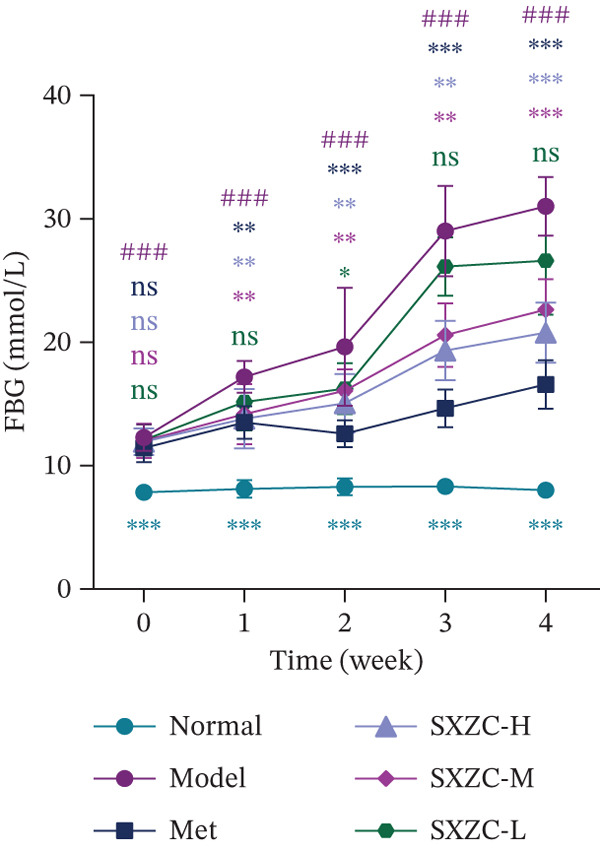
(a)

**Figure:**
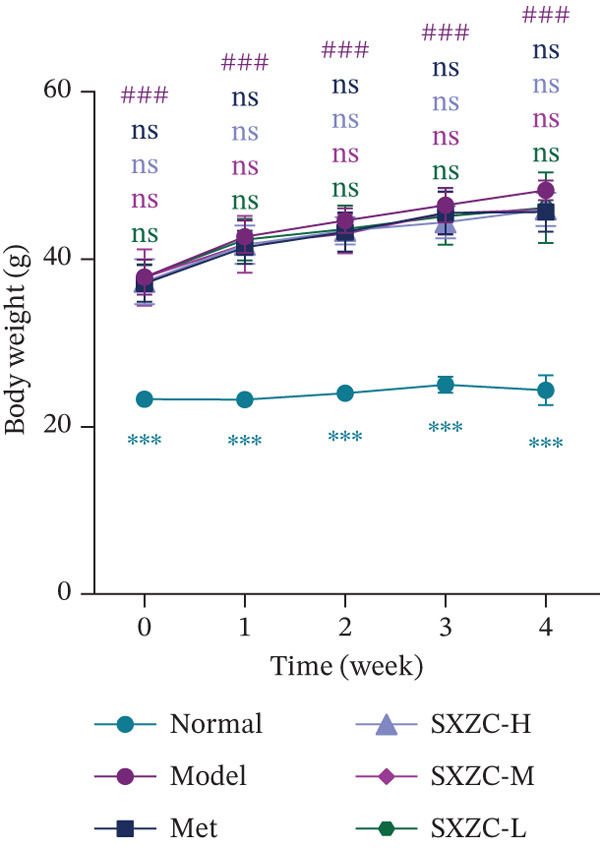
(b)

**Figure:**
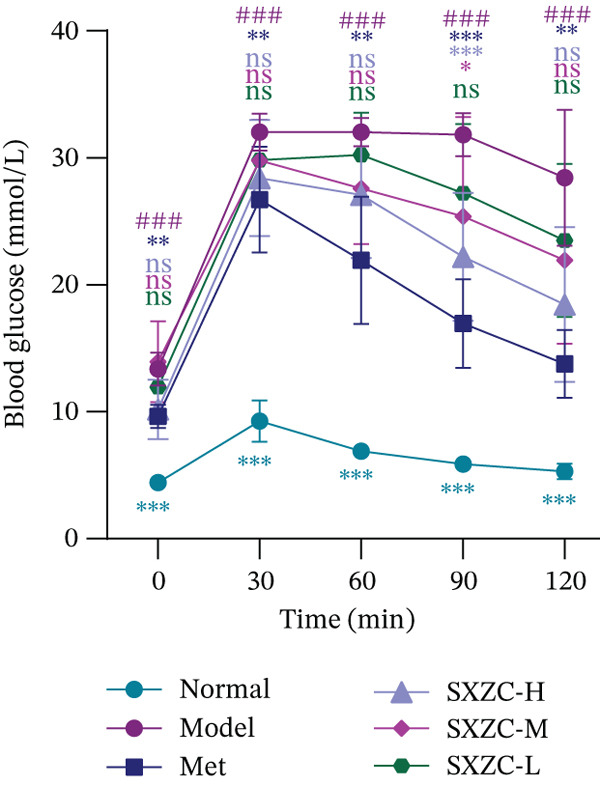
(c)

**Figure:**
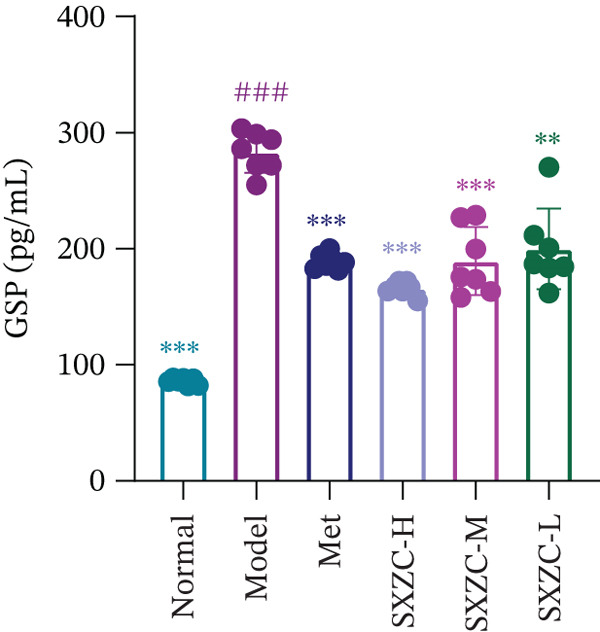
(d)

**Figure:**
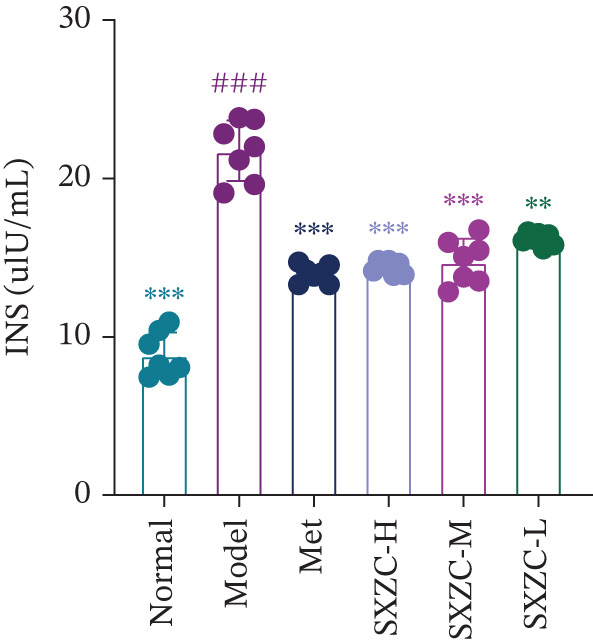
(e)

**Figure:**
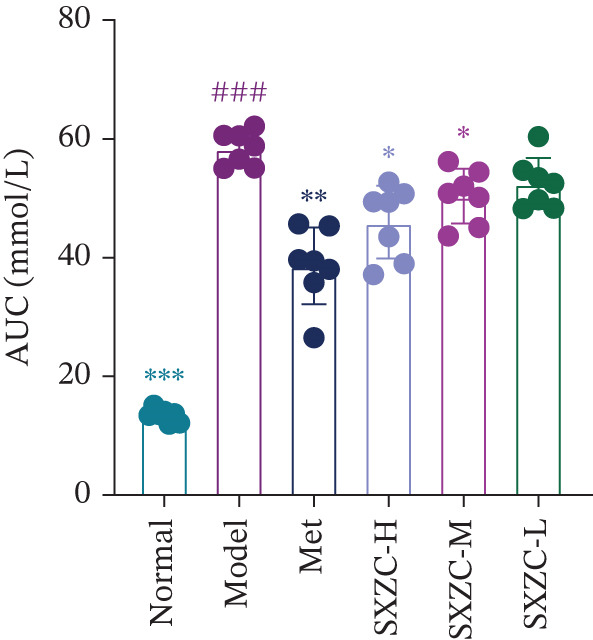
(f)

### 3.2. SXZC Formula Inhibits Inflammation and Increases the Content of Glycogen Granules in the Liver

To further investigate the effect of SXZC formula on hepatic glucose metabolism, we performed HE staining and PAS staining on pathological sections of the liver and measured the levels of IFs in it. HE staining (Figure 2a) showed that the model group had structural disorders of the hepatic lobules, dilatation of the central vein, and ballooning degeneration of the hepatocytes, which accompanied with a large number of lipid vacuoles and inflammatory infiltration. Compared with the model group, the Met and all SXZC‐treated groups showed notable improvement in the aforementioned pathological changes. PAS staining (Figure 2b) revealed a marked reduction in diffusely distributed *β* particles and clumped *α* particles within hepatocytes of the model group. Following the administration of Met or SXZC formula, numerous scattered purple *β* particles and purplish‐red aggregates formed by clustered *α* particles became visible within cells. These demonstrate that Met and SXZC formula effectively promote hepatic glycogen synthesis and storage. In addition, the results of hepatic tissue IF assay (Figures 2c, 2d, and 2e) showed that both Met and SXZC formula reduced hepatic TNF‐*α*, IL‐6, and IL‐1*β* levels compared with the model group. Furthermore, the improvement in IFs and glycogen content appeared to be more pronounced with increasing doses of SXZC formula.

Figure 2SXZC formula inhibits inflammation and increases content of glycogen granules in the liver. (a) HE staining of pathological sections of liver from each group (20×). Inflammatory infiltration and steatosis were reduced in SXZC‐administered drugs compared with the model group. Red arrows indicate inflammatory infiltrates. (b) PAS staining of pathological sections of livers from each group (20×). (c) TNF‐*α* levels in the liver tissue of mice (*n* = 7) in every group. (d) IL‐6 levels in the liver tissue of mice (*n* = 7) in every group. (e) IL‐1*β* levels in the liver tissue of mice (*n* = 7) in every group. Met: metformin, SXZC‐H: high dose of SXZC formula, SXZC‐M: medium‐dose of SXZC formula, SXZC‐L: low‐dose of SXZC formula. Compared with the normal group, #*p* < 0.05, ##*p* < 0.01, ###*p* < 0.001, Compared with the model group,  ^∗^ *p* < 0.05,  ^∗∗^ *p* < 0.01,  ^∗∗∗^ *p* < 0.001.

**Figure:**
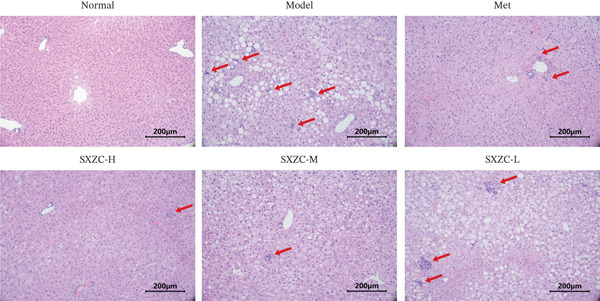
(a)

**Figure:**
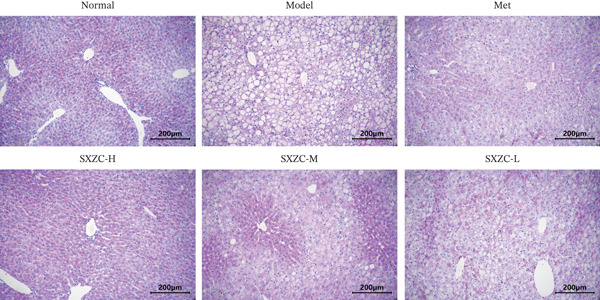
(b)

**Figure:**
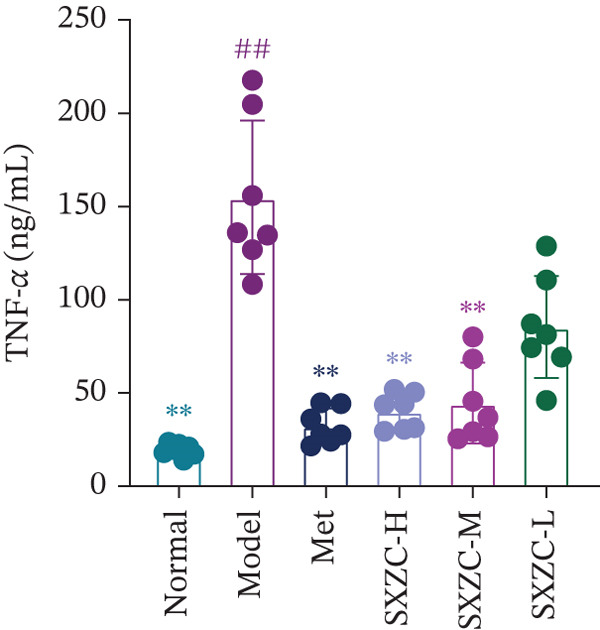
(c)

**Figure:**
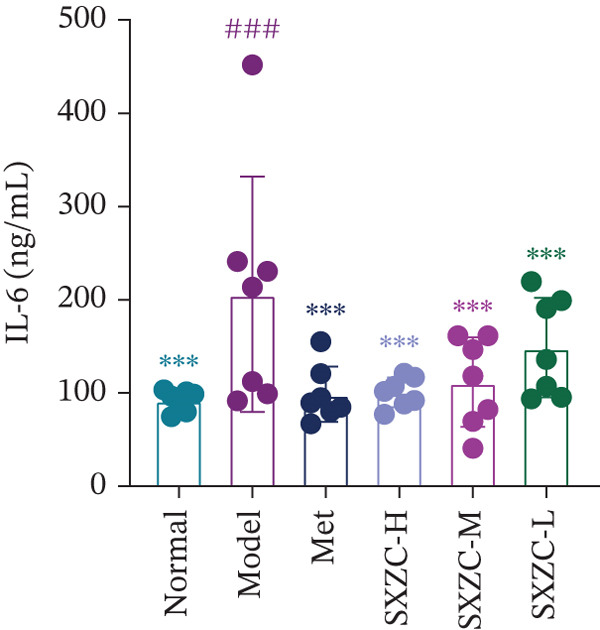
(d)

**Figure:**
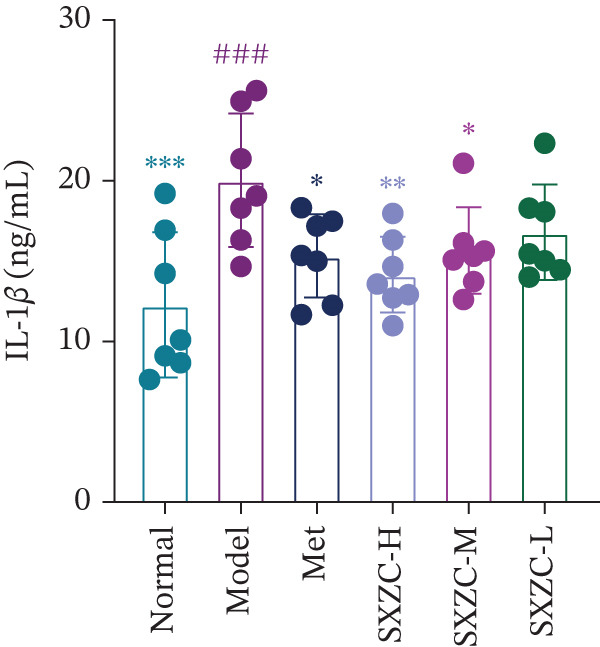
(e)

### 3.3. The JNK Signaling Pathway Serves as a Potential Pathway for the SXZC Formula

To explore the molecular mechanisms by which SXZC formula inhibits inflammation and increases glycogen content, we performed a network pharmacological analysis. Five hundred and ninety‐two possible targets of SXZC formula and 6412 targets related to T2DM were identified from the database. Subsequent analysis using Venny 2.1.0 yielded 295 disease‐prescription cross‐targets (Figure 3a). Using STRING and Cytoscape software, we constructed a PPI network, obtained core targets (Figure 3b) and potential targets (Figure 3c) based on PPIs, and identified five core targets (TNF, AKT1, EGFR, IL1B, and SRC) associated with the treatment of T2DM. With Cytoscape, we constructed a disease formula–ingredient target–interaction map. It indicates that the ingredients of *American ginseng* and *Anemarrhenae Rhizoma* contributed a substantial proportion to the formula′s efficacy. The BP of GO are related to cell motility, mitogen‐activated protein kinase (MAPK) cascade regulation, and receptor/kinase signaling pathways (Figure 3g). The MF involved the regulation of protein kinase, phosphatase, and serine/threonine kinase activities. The main signaling pathways of KEGG relevant to T2DM include PI3K‐Akt signaling pathway, endocrine resistance, MAPK signaling pathway, rat sarcoma virus (Ras) signaling pathway, and Ras‐related protein 1 (Rap1) signaling pathway (Figure 3e,f). Among these, the MAPK signaling pathway was selected for further investigation. JNK1 (Mapk8) could serve as a critical link between inflammatory response and glycogen synthesis (Figure 3h,i).

Figure 3The JNK signaling pathway serves as a potential pathway for the SXZC formula. (a) Venny diagram of SXZC formula active ingredient predicted targets versus T2DM disease, with a total of 295 intersecting targets. (b) PPI plot of core targets. (c) PPI plot of potential targets. (d) Disease–SXZC–ingredient‐target network graph. It can be seen that *American ginseng* (AGS) and *Anemarrhenae Rhizoma* (AR) are the main sources of potency. RR represents *Rehmanniae Radix*, and ATR represents *Rhizoma Atractylodis.* (e) Bubble graph of KEGG enrichment, (f) KEGG enrichment histogram. (g) Bar graph of GO enrichment, including BP, CC, and MF. (h) Intersecting targets in the MAPK pathway, with JNK/c‐Jun cascade in the red box. (i) Molecular pathway by which JNK1 affects glycogen synthesis in the insulin resistance pathway. Color shades and node sizes correlate with the degree under each target (b–d).

**Figure:**
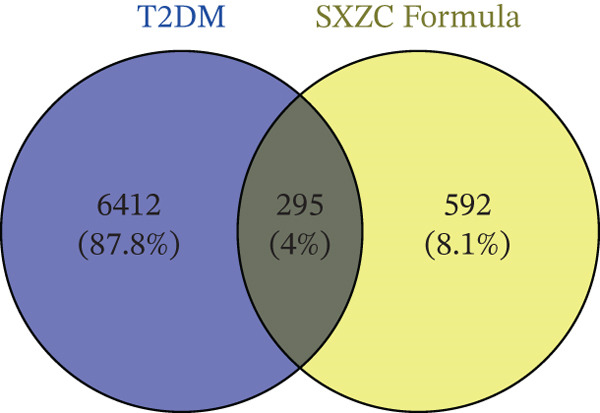
(a)

**Figure:**
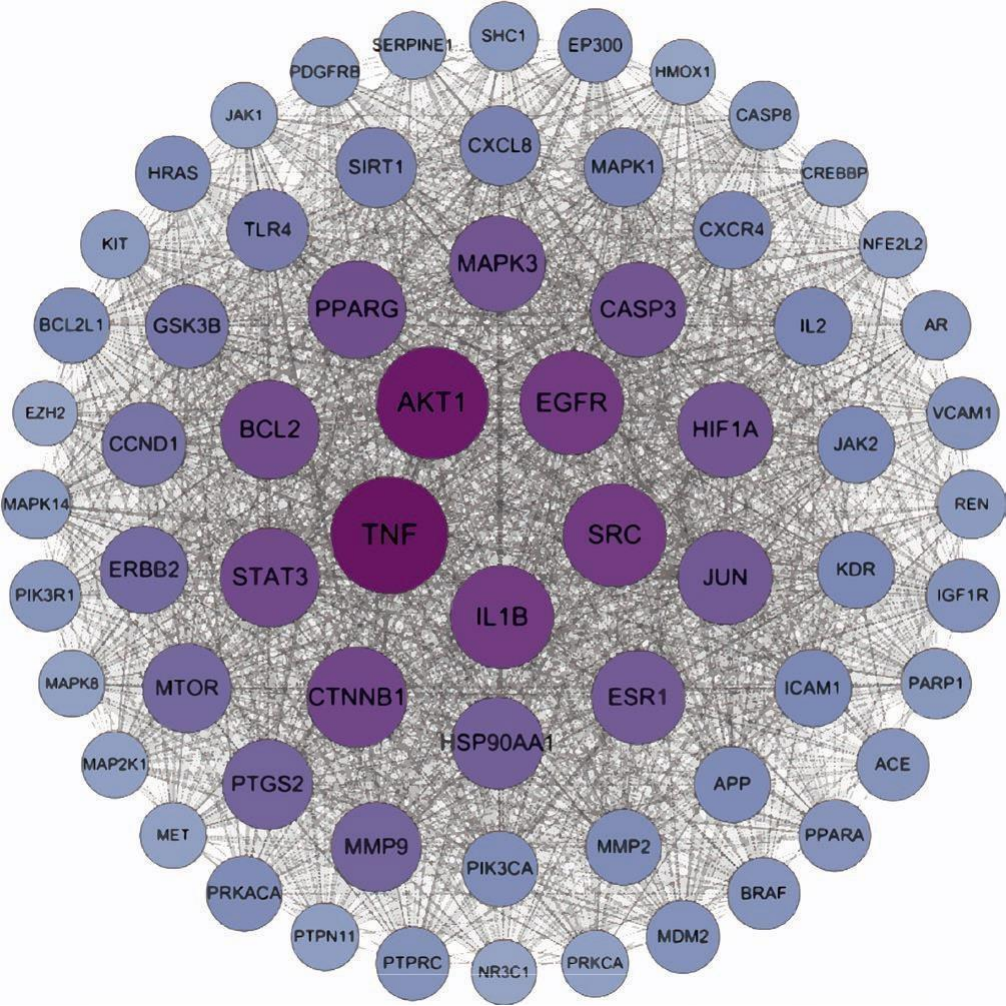
(b)

**Figure:**
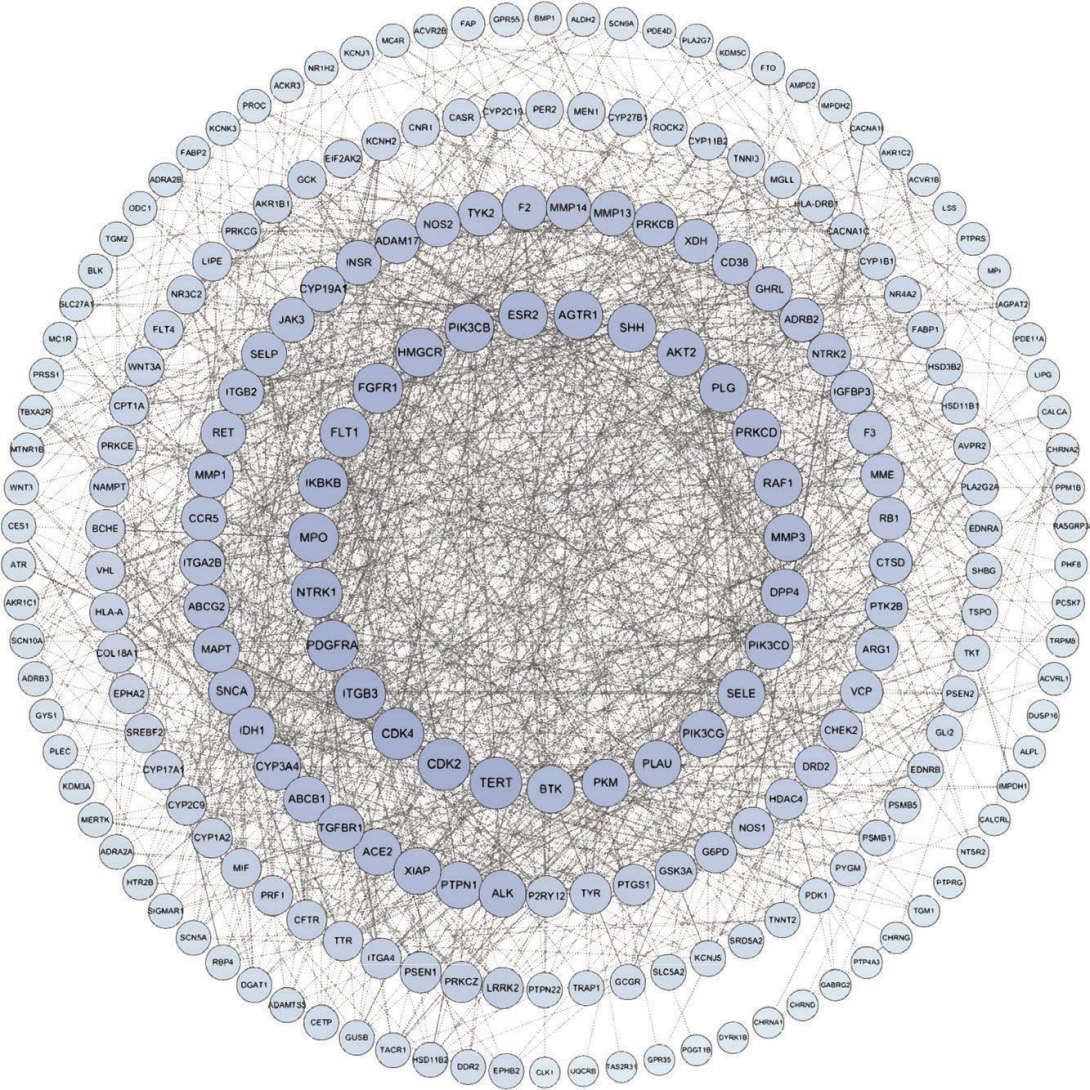
(c)

**Figure:**
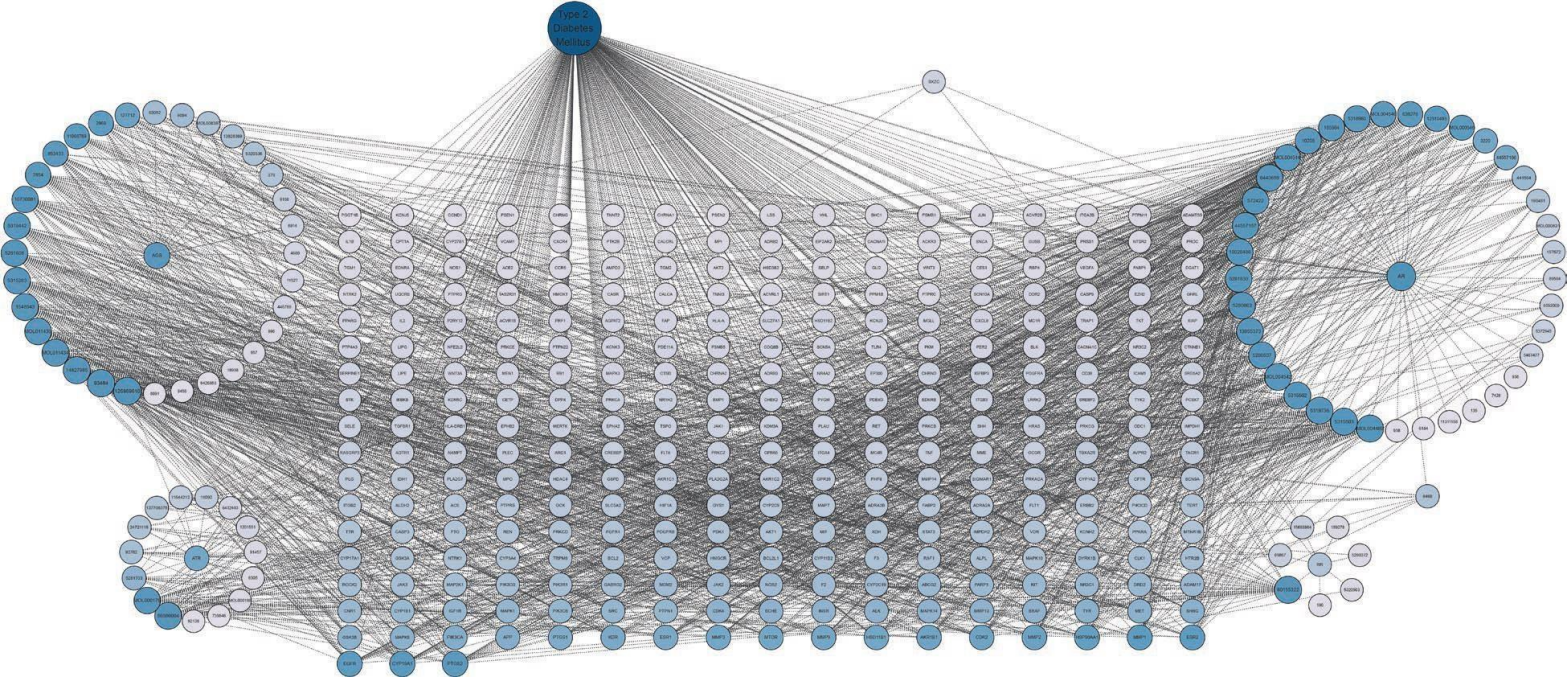
(d)

**Figure:**
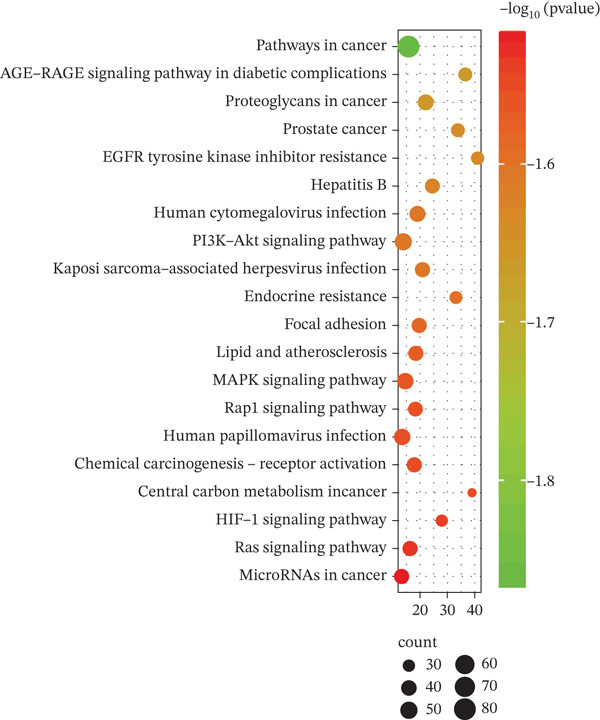
(e)

**Figure:**
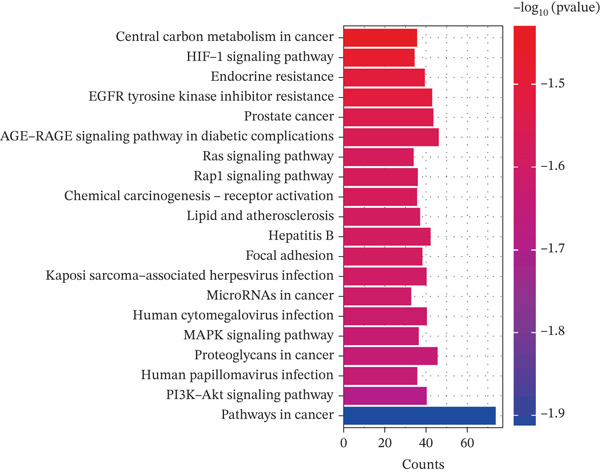
(f)

**Figure:**
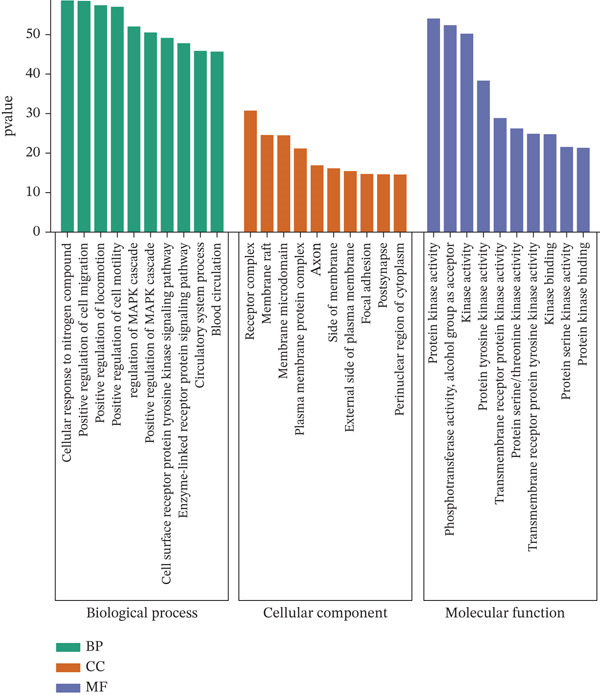
(g)

**Figure:**
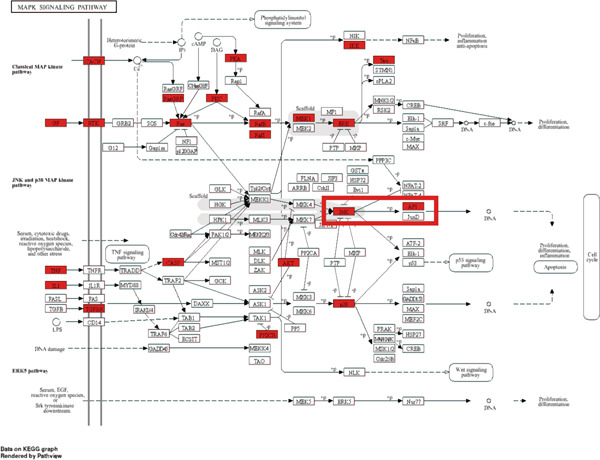
(h)

**Figure:**
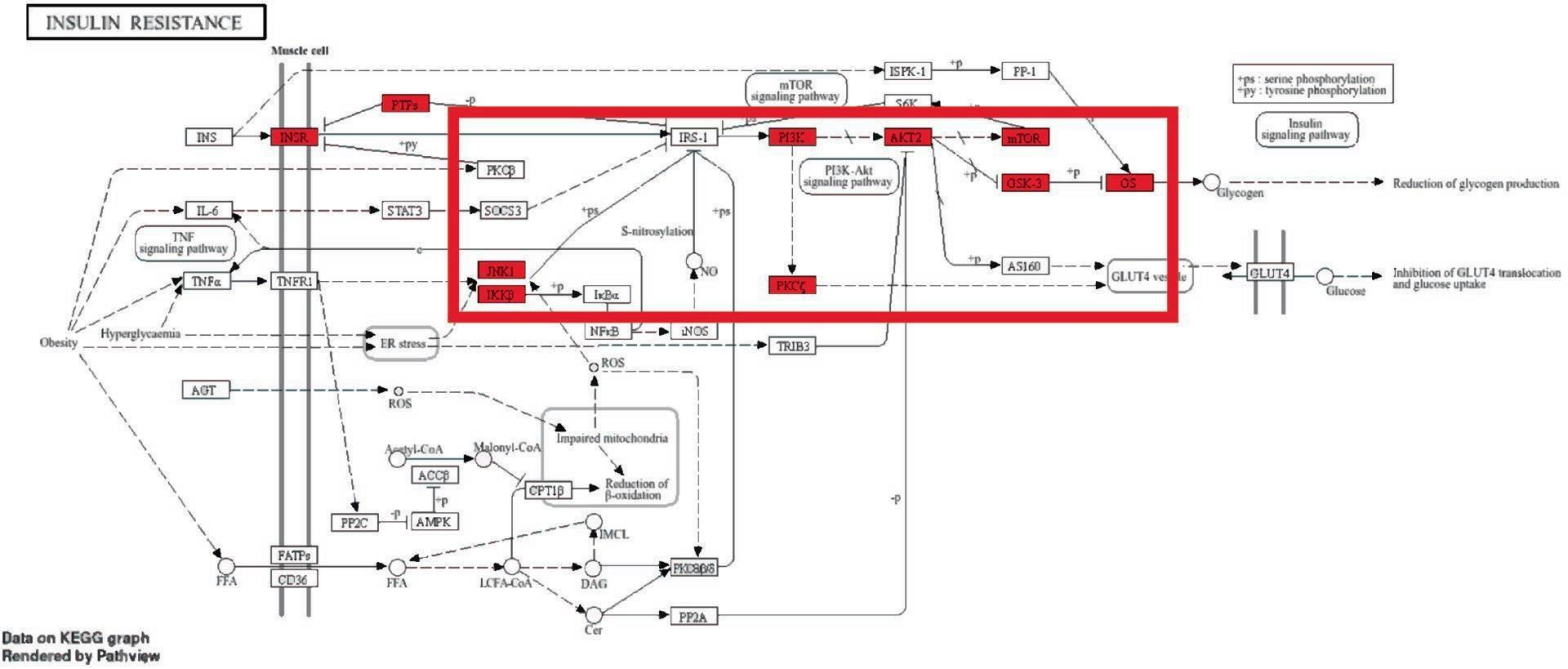
(i)

### 3.4. Main Components of SXZC Formula Bind Tightly With JNK1

To investigate whether the active ingredients in SXZC formula could interact with Mapk8, we conducted molecular docking to validate the interaction. Fourteen ingredients may interplay with JNK1. Molecular docking demonstrated that the docking binding energies were all below −4.5 kcal/mol, suggesting stable binding and a high likelihood of in vivo (Table 2). The complexes with binding energies less than −7 kcal/mol were observed and analyzed. The ligands primarily formed hydrogen bonds with key amino acid residues in the Mapk8, including SER34, SER155, and ASN114. Three ligands with the lowest binding energies were bound in the adenosine triphosphate (ATP)–competitive binding cleft of Mapk8 (Figure 4a, 4b, 4c, and 4d). Then we performed 100‐ns molecular dynamics simulations to analyze its stability, choosing the protein–ligand complex with the lowest binding energy (Figure 4j). RMSD showed low overall fluctuations in the complex throughout the simulation, indicating a stable complex between aurantiamide and Mapk8. Besides, it suggests that aurantiamide may act as a potential ATP‐competitive inhibitor of JNK1 kinase activity. Analysis of the protein backbone RMSF (Figure 4k) showed that SER 34 and SER 155 (binding sites) were more flexible than the surrounding residues, suggesting that aurantiamide may bind Mapk8 at these sites.

**Table 2:** Docking binding energy of the active ingredient of the compound to the JNK1 molecule.

Target	Ligands	Source of botanical medicine	Binding energy (kcal/mol)	Hydrogen bonds
Mapk8	Aurantiamide	*Anemarrhenae Rhizoma*	−9.2	SER 34, SER 155
	[(2S)‐2‐[[(2R)‐2‐benzamido‐3‐phenylpropanoyl] amino]‐3‐phenylpropyl] acetate	*Anemarrhenae Rhizoma*	−9.1	SER 34, ASN 156, SER 155, ASN 114
	Asperglaucide	*Anemarrhenae Rhizoma*	−9.0	GLY 35, SER 34, ASN 114
	Broussonin A	*Anemarrhenae Rhizoma*	−8.2	LYS 55
	Diosgenin	*Anemarrhenae Rhizoma*	−7.9	ASN 205
	Anemarsaponin F_qt	*Anemarrhenae Rhizoma*	−7.9	GLN 117
	Diincarvilone A	*Rehmanniae Radix*	−7.6	S299, L241
	(9S,10R)‐10‐(acetyloxy)‐8,8‐dimethyl‐2‐oxo‐9H,10H‐pyrano[2,3‐h] chromen‐9‐yl 2‐methylbut‐2‐enoate	*American Ginseng*	−7.4	ASN 287
	Hippeastrine	*Anemarrhenae Rhizoma*	−7.4	SER 34, SER 155, GLN 37, ARG 69
	2‐hydroxyisoxypropyl‐3‐hydroxy‐7‐isopentene‐2,3‐dihydrobenzofuran‐5‐carboxylic	*Rhizoma Atractylodis*	−6.8	ARG 69, THR 65
	Timosaponin B III_qt	*Anemarrhenae Rhizoma*	−6.8	GLN 62
	Panaxytriol	*American Ginseng*	−6.6	MET 111
	Linarionoside A	*American ginseng*	−6.5	LYS 203
	PQ‐2	*American ginseng*	−6.4	ASN 114, SER 34, ARG 69

Figure 4Main components of SXZC formula bind tightly with JNK1. Molecular docking models of JNK1 with mainly ingredients in SXZC formula, where the structure in yellow is the active ingredient, the structure in purple is the amino acid residues in the JNK1 docking site, and the yellow dashed line is the hydrogen bond connecting the ligand to the protein. (a) Aurantiamide, (b) [(2S)‐2‐[[[2R]‐2‐benzamido‐3‐phenylpropanoyl] amino]‐3‐phenylpropyl] acetate, (c) asperglaucide, (d) broussonin A, (e) anemarsaponin F qt, (f) diosgenin, (g) diincarvilone A, (h) (9S,10R)‐10‐(acetyloxy)‐8,8‐dimethyl‐2‐oxo‐9H,10H‐pyrano [2,3‐h] chromen‐9‐yl 2‐methylbut‐2‐enoate, (i) hippeastrine. (j) 0–100‐ns RMSD analysis of kinetic simulations of aurantiamide and Mapk8 complexes to assess complex binding tightness. (k) RMSF during kinetic simulation of aurantiamide and Mapk8 complexes to assess the local conformational changes of the protein.

**Figure:**
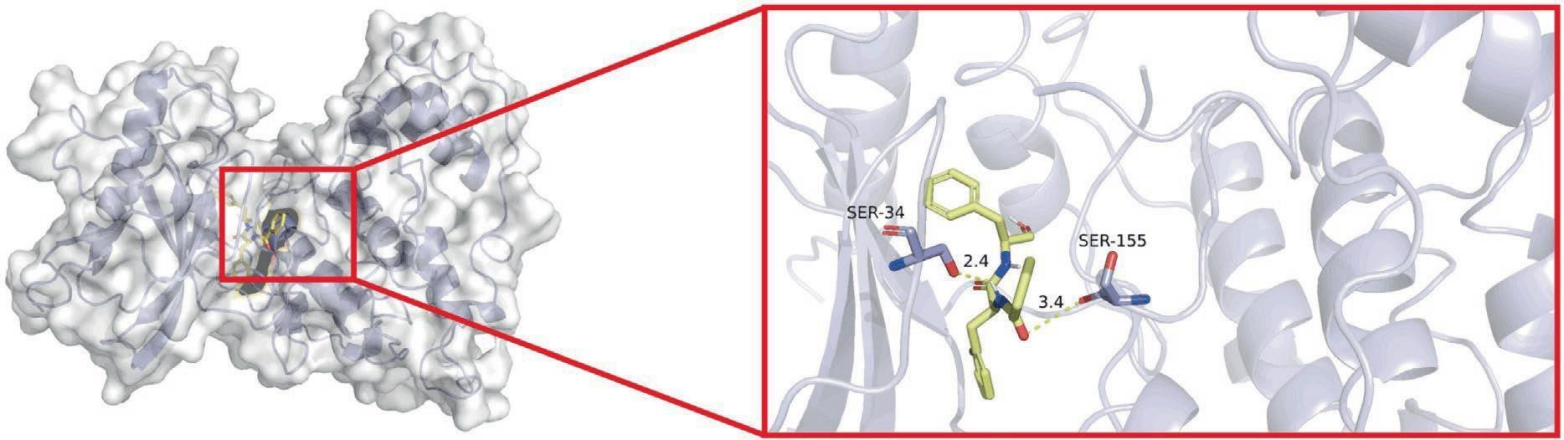
(a)

**Figure:**
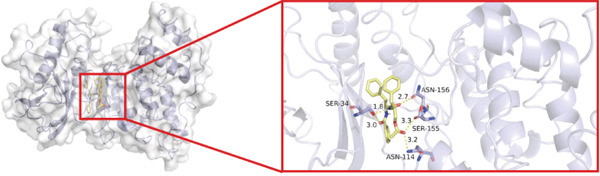
(b)

**Figure:**
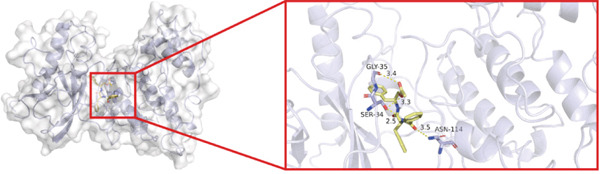
(c)

**Figure:**
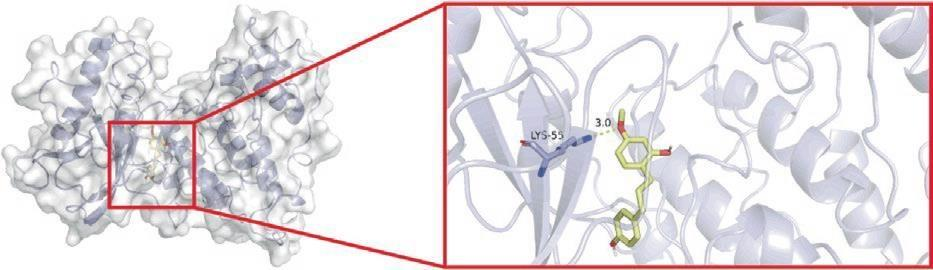
(d)

**Figure:**
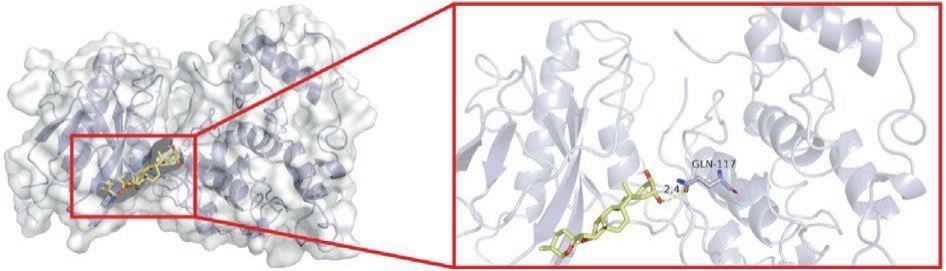
(e)

**Figure:**
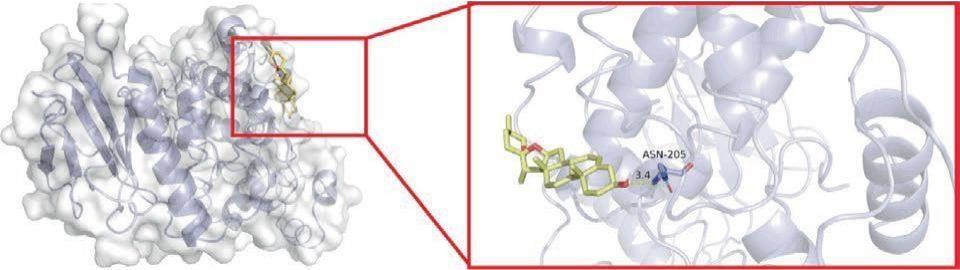
(f)

**Figure:**
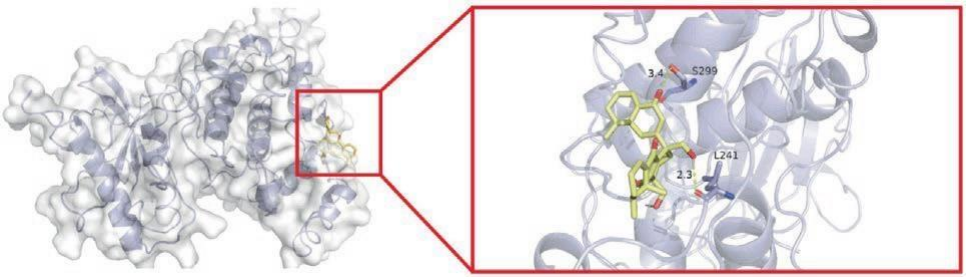
(g)

**Figure:**
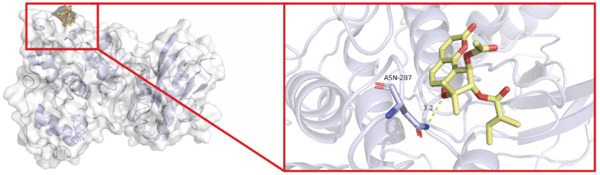
(h)

**Figure:**
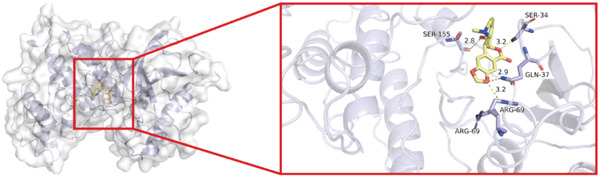
(i)

**Figure:**
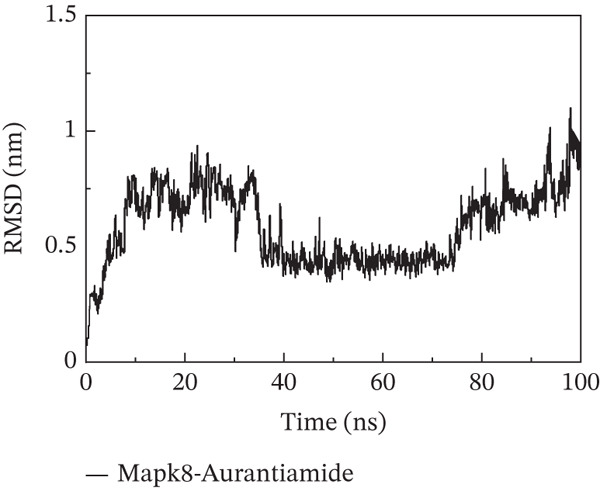
(j)

**Figure:**
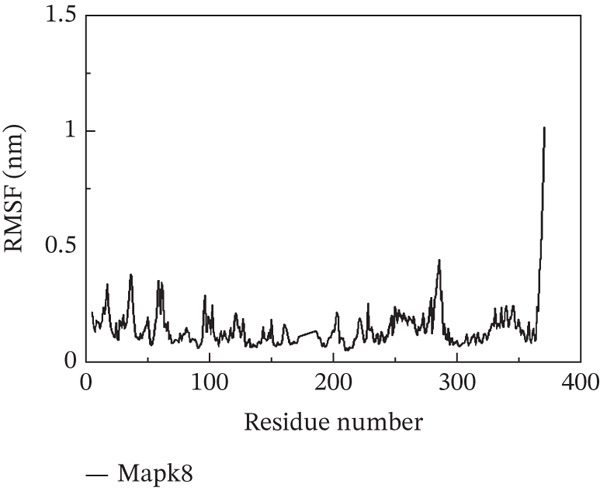
(k)

### 3.5. SXZC Formula Promotes Hepatic Glycogen Synthesis and Inhibits Inflammation Through the JNK/c‐Jun/IRS1/GSK3*β* Signaling Pathway

We assessed the activity of key signaling molecules in the proposed pathway by measuring their phosphorylation levels via WB. The results showed a significant decrease in the phosphorylation levels of JNK, c‐Jun, and IRS1 (Figures 5a, 5b, and 5c), and a significant increase in the inhibitory phosphorylation of GSK3*β* (Figure 5d), which would promote glycogen synthesis. Furthermore, we determined the expression levels of glucose‐6‐phosphatase catalytic subunit 1 (G6pc1) and phosphoenolpyruvate carboxy kinase 1 (Pck1), which are the rate‐limiting enzymes in gluconeogenesis. The expression levels of both G6pc1 and Pck1 were significantly lower compared with the model group (Figure 5e,f). These findings suggest that the SXZC formula promotes hepatic glycogen synthesis by the JNK/c‐Jun/IRS1/GSK3*β* signaling pathway, ultimately improving glucose homeostasis.

Figure 5SXZC formula promotes hepatic glycogen synthesis and inhibits inflammation through the JNK/c‐Jun/IRS1/GSK3*β* signaling pathway. (a) Changes in phosphorylation level of JNK in each group (*n* = 3). (b) Changes in phosphorylation level of c‐Jun in each group (*n* = 3). (c) Changes in phosphorylation level of IRS1 in each group (*n* = 3). (d) Changes in phosphorylation level of GSK3*β* in each group (*n* = 3). (e) Changes in relative expression of G6pc1 in each group (*n* = 5). (f) Changes in relative expression of Pck1 in each group (*n* = 5). Met: metformin group, SXZC‐H: high dose of SXZC formula, SXZC‐M: medium dose of SXZC formula, SXZC‐L: low dose of SXZC formula. Compared with the model group,  ^∗^ *p* < 0.05,  ^∗∗^ *p* < 0.01,  ^∗∗∗^ *p* < 0.001.

**Figure:**
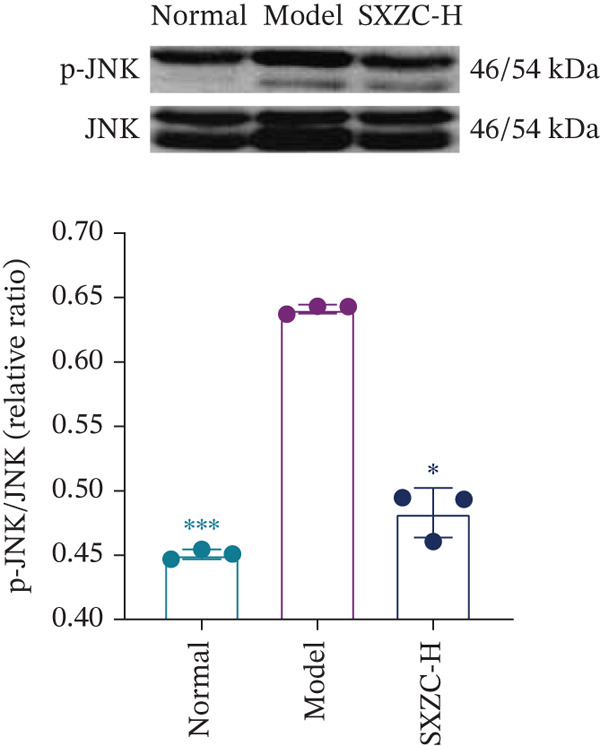
(a)

**Figure:**
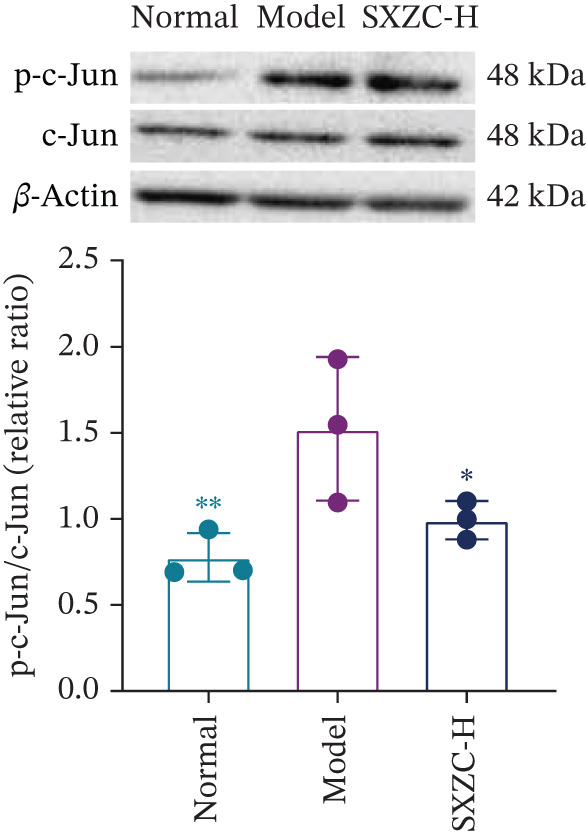
(b)

**Figure:**
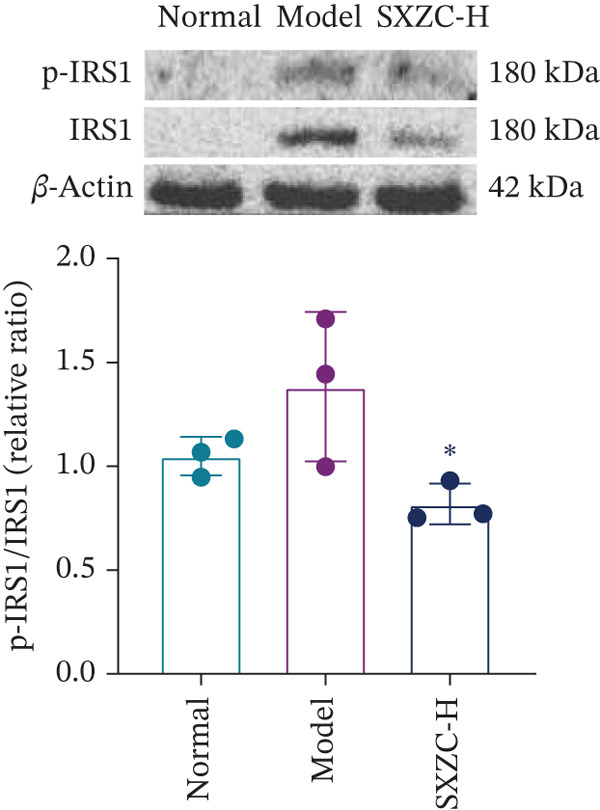
(c)

**Figure:**
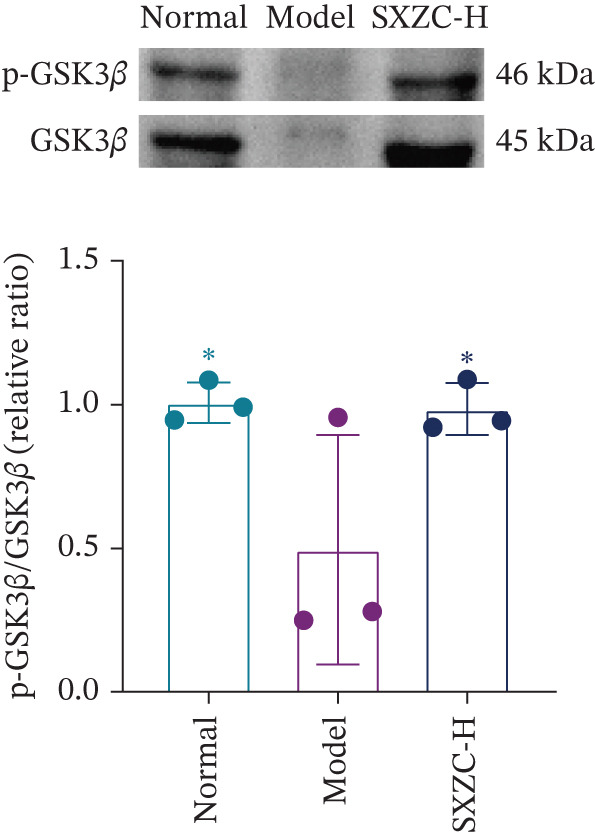
(d)

**Figure:**
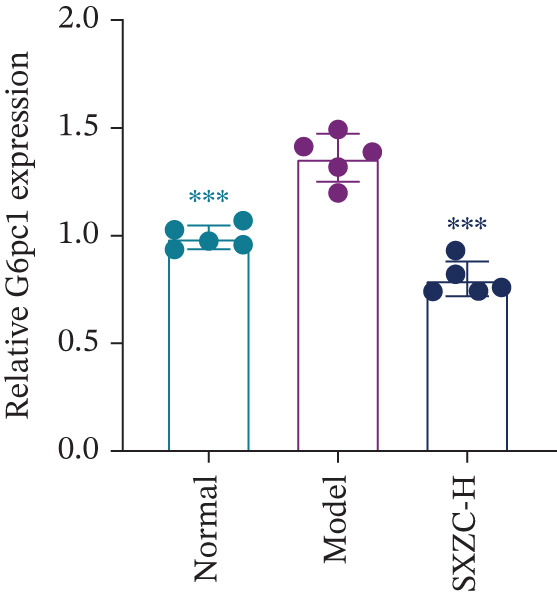
(e)

**Figure:**
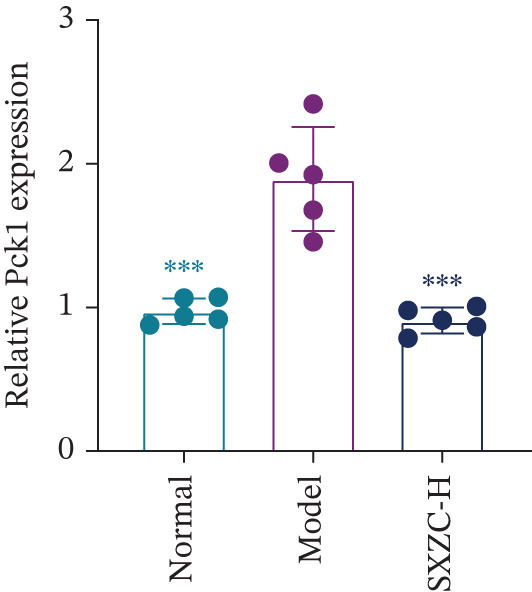
(f)

## 4. Discussion

Hepatic glycogen synthesis plays a critical role in maintaining glucose homeostasis [2], and the inhibition of it mediated by the inflammatory causes persistent hyperglycemia in diabetes [9]. Amelioration of the inflammation‐induced impairment of liver glucose metabolism is one of the therapeutic approaches for DM.

In the theory of TCM, the etiology of DM is mainly due to yin deficiency, internal heat [31], and fluid depletion caused by a variety of factors (genetics, diet, and/or lifestyles). *Rehmanniae Radix*, *American ginseng*, *Anemarrhenae Rhizoma*, and *Rhizoma Atractylodis* exert efficacy in clearing heat, nourishing yin, generating fluids, fortifying the spleen, and drying dampness. They have been widely used in the treatment of diabetes since ancient times [32]. Formulating them into a combination at a 3:1:2:1 ratio in accordance with the TCM theories embodies the holistic approach to disease treatment in TCM. The synergistic effects among herbs provide advantages for the treatment of complex metabolic disorders [11]. To investigate whether the SXZC formula could ameliorate blood glucose levels via hepatic glucose metabolism, we administered it to db/db mice for a 4‐week duration. Subsequent experiments validated our hypothesis.

Significant and sustained elevation of blood glucose levels after oral glucose administration was observed in OGTT, and the contrast in AUC indicated improved INS sensitivity after SXZC formula treatment. Also, FBG levels and serum levels of GSP and INS measured weekly indicated the role of SXZC formula in maintaining glycemic homeostasis. To visualize the pathological changes in the liver, we applied PAS staining and HE staining. The results showed a significant increase in the content of glycogen particles after the duration, as well as an improved hepatic inflammatory infiltrate observed. To clarify this alteration even more, we measured the levels of major IFs in the liver. The outcomes suggest that SXZC formula suppressed hepatic inflammation in a dose‐dependent manner. Combined with the phenotypic changes in the animals, the hypothesis is proposed that the hypoglycemic effect of SXZC formula is achieved by ameliorating the hepatic inflammatory response and promoting hepatic glycogen synthesis.

In order to explore the potential molecular mechanisms of SXZC formula, we introduced network pharmacology to quantify the synergistic effects among herbal medicines and construct a “component target–pathway” network to translate the efficacy of herbal compounding into a rational scientific explanation. Based on databases, we screened and obtained 295 disease‐complex intersection targets. PPI analysis showed that TNF, AKT1, EGFR, IL1B, and SRC are the key targets, which focus on inflammation and metabolism regulation [33–37]. The finding was highly compatible with our animal phenotypes. The GO and KEGG enrichment analyses further revealed that the SXZC formula might be mediated by the PI3K‐Akt signaling pathway, endocrine resistance, MAPK signaling pathway, Ras signaling pathway, and Rap1 signaling pathway, which are several molecular pathways associated with the development of T2DM. The weakening of PI3K/AKT pathway signaling could trigger the reduction of glycogen storage in the liver [38]. The abnormal activation of the Ras pathway will disrupt glucose metabolism through the modulation of the downstream targets of the ERK1/2 and PI3K/AKT pathways [39]. Activation of Rap1A/phospholipase C‐*ε* (PLC‐*ε*) signaling will promote the production of diacylglycerol (DAG) and inositol trisphosphate (IP3) synthesis, resulting in the abnormal breakdown of INS granules. Endocrine resistance is a larger network of relationships, of which the IR pathway is a branch [40]. The MAPK signaling pathway responds through a cascade initiated by four MAPK isoforms: extracellular‐regulated kinase 1/2 (ERK1/2), JNK1/2/3, p38 MAPK (p38), and ERK5. Aberrant activation of the JNK exacerbates the hepatic inflammatory response by phosphorylation of c‐Jun, while directly inhibiting phosphorylation of IRS1 (ser307), blocking PI3K/AKT2 signaling, leading to reduced hepatic glycogen synthesis [39, 41]. It indicates that the MAPK pathway acts as a link between inflammation and glycogen synthesis, and JNK isoforms are the primary candidates for SXZC formula action.

In order to validate the network pharmacological predictions, we applied molecular docking and molecular dynamics simulation to observe the stability of protein–ligand binding and the site of action. Fourteen compounds in SXZC formula could bind to JNK1 tightly, and four of the high‐affinity components target the ATP‐binding cleft of JNK1, which is a key region of action for ATP‐competitive inhibitors [42]. RMSD in a 100 ns kinetic simulation showed that the protein–ligand complex was lower than 3 Å, and the RMSF characteristics of the binding site were consistent with the ligand recognition pattern [43, 44]. These represent that the components in SXZC formula could bind to JNK1 and exert an inhibitory effect.

We delve into the effects of SXZC formula on the expression of key proteins in the pathway, including JNK, c‐Jun, IRS1, and GSK3*β*. In terms of inflammation regulation, the phosphorylation levels of JNK and c‐Jun were significantly reduced in the liver after SXZC formula intervention. It was consistent with the decreased IFs, confirming its inhibitory effect on the JNK/c‐Jun inflammatory pathway. As for hepatic glycogen synthesis, the phosphorylation of ser307 of IRS1 was reduced, and the phosphorylation of ser9 of GSK3*β* was increased, which directly promoted GS activity and hepatic glycogen synthesis. Glycogen biosynthesis would inhibit gluconeogenesis in turn [3], so we measured the transcript levels of G6pc1 and Pck1, finding that their expression was downregulated. These demonstrate that SXZC formula ameliorates hepatic glucose metabolism disorders by regulating the JNK/c‐Jun/IRS1/GSK3*β* pathway.

Nevertheless, network pharmacology, molecular docking, and molecular dynamics simulations rely on data and algorithms, and the results might be different from the actual results due to database and software limitations. Although we performed the validation, it does not represent all the real effects of the SXZC formula components in the body.

## 5. Conclusion

**Figure 6:**
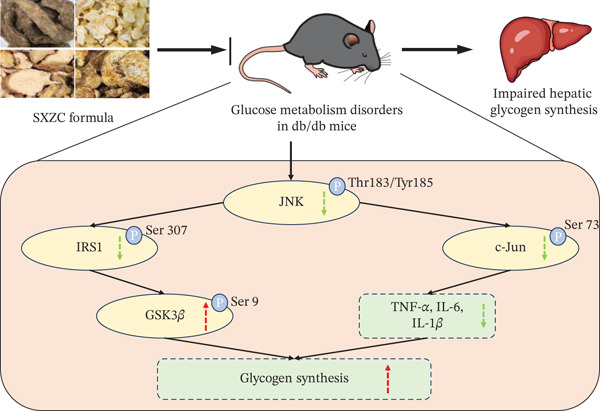
The mechanism of SXZC formula in producing a hypoglycemic effect. Green arrows represent decreases, red arrows represent increases, and P represents phosphorylation and site. SXZC formula reduces inflammatory factor levels by inhibiting JNK/c‐Jun/IRS1 phosphorylation and promotes GSK3*β* phosphorylation, resulting in enhanced glycogen synthesis and reduced inflammatory response.

In conclusion, our research confirmed the role of SXZC formula in alleviating hepatic inflammation and promoting hepatic glycogen synthesis by the JNK/c‐Jun/IRS1/GSK3*β* signaling pathway (Figure 6). This reveals the potential of SXZC formula in the treatment of diabetes.

**Nomenclature**
DM: diabetes mellitus
IR: insulin resistance
IFs: inflammatory factors
TNF‐*α*
: tumor necrosis factor‐*α*
IL‐1*β*
: interleukin 1 *β*
IL‐6: interleukin‐6
JNK: c‐Jun N‐terminal kinase
PI3K: phosphatidylinositol 3‐kinase
Akt: protein kinase B
GSK3: glycogen synthase kinase 3
TCM: traditional Chinese medicine
SXZC: Shengdihuang Xiyangshen Zhimu Cangzhu
TRPV1: transient receptor potential cation channel subfamily V member 1
SCD1: stearoyl‐CoA desaturase 1
STZ: streptozotocin
AMPK: AMP‐activated protein kinase
FoxO1: Forkhead box protein O1
DGK*θ*
: diacylglycerol kinase *θ*
PGC1*α*
: peroxisome proliferator–activated receptor‐*γ* coactivator l *α*
UCP1: uncoupling protein 1
IRS1: insulin receptor substrate 1
GSK3*β*
: glycogen synthase kinase‐3 *β*
FBG: fasting blood glucose
OGTT: oral glucose tolerance test
AUC: area under the curve
INS: insulin
GSP: glycated serum protein
ELISA: enzyme‐linked immunosorbent assay
OD: optical density
HE: hematoxylin–eosin staining
PAS: periodic acid‐Schiff staining
RT‐qPCR: reverse transcription‐quantitative polymerase chain reaction
RNA: ribonucleic acid
cDNA: complementary deoxyribonucleic acid
WB: Western blot
T2DM: Type 2 diabetes mellitus
GO: gene ontology
BP: biological process
MF: molecular function
CC: cellular component
KEGG: Kyoto Encyclopedia of Genes and Genomes
PPI: protein–protein interaction
RMSD: root mean square deviation
RMSF: root mean square fluctuation
ANOVA: one‐way analysis of variance
LSD: Fisher′s least significant difference
TNF: tumor necrosis factor
AKT1: protein kinase B 1
EGFR: epidermal growth factor receptor
IL1B: interleukin 1 β
SRC: steroid receptor coactivator
MAPK: mitogen‐activated protein kinase
Ras: rat sarcoma virus
Rap1: Ras‐related protein 1
JNK1: c‐Jun N‐terminal kinase 1
Mapk8: mitogen‐activated protein kinase 8
G6pc1: glucose‐6‐phosphatase catalytic subunit 1
Pck1: phosphoenolpyruvate carboxykinase 1
ERK1/2: extracellular‐regulated kinase 1/2
PLC‐*ε*
: phospholipase C‐*ε*
DAG: diacylglycerol
IP3: inositol trisphosphate
p38: p38 mitogen‐activated protein kinase

## Author Contributions

Paper writing by Kewen Qu, Mei Li, and Shangping Wu; experimental program and writing review by Xiangyu Guo, Shuxin Wu, and Mei Li; experimental work by Kewen Qu, Jinxu Sun, Xinyu Liu, Kanglin Liu, and Shangping Wu; data collection and analysis by Kewen Qu.

## Funding

This study was supported by the National Natural Science Foundation of China (82174351) and Beijing Economic Development Zone Yicheng Talent Project (2024‐YCRC‐068).

## Disclosure

All authors approved the final version of the manuscript.

## Ethics Statement

The animal experiments in this experiment have been ethically applied for and approved in accordance with the normative requirements (Approval No. 2024071104‐3260) and the experimental procedures were conducted in strict compliance with the ethical requirements.

## Conflicts of Interest

The authors declare no conflicts of interest.

## Data Availability

The data and graphs in this experiment can be shared upon reasonable request to the authors of the article.
